# Identification and age-dependence of pteridines in bed bugs (*Cimex lectularius*) and bat bugs (*C. pipistrelli*) using liquid chromatography-tandem mass spectrometry

**DOI:** 10.1038/s41598-020-66919-5

**Published:** 2020-06-23

**Authors:** Jana Křemenová, Ondřej Balvín, Oliver Otti, Michal Pavonič, Klaus Reinhardt, Zdeněk Šimek, Tomáš Bartonička

**Affiliations:** 1 Masaryk University, Faculty of Sciences, Department of Botany and Zoology, Brno, 61137 Czech Republic; 2 Czech University of Life Sciences Prague, Faculty of Environmental Science, Department of Ecology, Prague, 16521 Czech Republic; 3 Universität Bayreuth, Animal Ecology I, Animal Population Ecology, Bayreuth, 95440 Germany; 4 Technische Universität Dresden, Department of Biology, Applied Zoology, Dresden, 01069 Germany; 5 Masaryk University, Research Centre for Toxic Compounds in the Environment, Brno, 62500 Czech Republic

**Keywords:** Biochemistry, Biological techniques, Ecology, Zoology

## Abstract

Determining the age of free-living insects, particularly of blood-sucking species, is important for human health because such knowledge critically influences the estimates of biting frequency and vectoring ability. Genetic age determination is currently not available. Pteridines gradually accumulate in the eyes of insects and their concentrations is the prevailing method. Despite of their stability, published extractions differ considerably, including for standards, for mixtures of pteridines and even for light conditions. This methodological inconsistency among studies is likely to influence age estimates severely and to hamper their comparability. Therefore we reviewed methodological steps across 106 studies to identify methodological denominators and results across studies. Second, we experimentally test how different pteridines vary in their age calibration curves in, common bed (*Cimex lectularius*) and bat bugs (*C. pipistrelli*). Here we show that the accumulation of particular pteridines varied between a) different populations and b) rearing temperatures but not c) with the impact of light conditions during extraction or d) the type of blood consumed by the bugs. To optimize the extraction of pteridines and measuring concentrations, we recommend the simultaneous measurement of more than one standard and subsequently to select those that show consistent changes over time to differentiate among age cohorts.

## Introduction

The development of an accurate methodology for determining the age of insects is a key technique to understanding many aspects of insect ecology, behaviour and effectiveness of pest control programmes. This particularly applies to blood-sucking, disease-transmitting insects where the age is an important parameter to predicting their biting frequency and hence vectoring ability^1^, as well as their fecundity^2^. Age-grading is also important in general evolutionary and ecological models that are based on insects, from individual to population level^3,4^. Finally, age-grading in insects has been important in veterinary and forensic medicine. Consequently, accurate methods for determining the age of adult insects in the field have gained considerable importance since the 60s of the 20th century^3,5,6^.

As all methods, currently available age-grading techniques also have limitations. Few genetic and cellular age markers exist and these, such as DNA methylation or telomere lengths, are related to cell division activity, and partially to age *per se*. Few markers are based on actual age, such as the circadian deposition of chitinous layers in cricket legs^7–10^. However, these circadian growth bands are visible till a certain age only and can vary with temperature^8,11^. Most markers indicate the accumulation of a marker with metabolic or other activity. For example, some markers are related to mandible abrasion with age and, therefore restricted to herbivorous insects^12^. Predictable anatomical changes in the reproductive system related to fertility changes, such as the accumulation of follicular relics and/or the appearance of ovarian tracheoles are also based on physiological age^5,13,14^. Finally, the standard method today appears to be a survey of the accumulation of fluorescent pigments in the eyes of insects^15,16^. It appears that most authors consider the deposition of pteridines as a chronological, rather than physiological age marker.

Most of the naturally occurring pteridines, called pterines, are the 2-amino-4-hydroxy derivatives^17,18^. Their stability allows for their long-term accumulation in the eye and minimal loss by decay^19^. Pteridines are poorly soluble in water, insoluble in non-polar organic solvents but soluble in strong acid or alkali. Other insect pigments, such as papiliochromes, anthocyanins and flavonoids are water-soluble^20,21^. The auto-chelating properties of pteridines result in high insolubility under normal physiological conditions and binding of the eye pteridines onto granules^15^. The pteridine content in both laboratory- and field-captured flies is typically a level of magnitude higher than the minimal detectable level and can be used to predict individual age in the laboratory population with high certainty^22^.

Pteridines have the advantage of being applicable to long-lived insects under different conditions and are measurable^23^. Found in the eyes, they are a group of fluorescent pigments derived from a pyrimidine-pyrazine ring structure. This structure is widely distributed in nature, being present in microorganisms, as well as plants and animals. In insects, pteridines have two important biological functions, i) as excretory products by inactivation of nitrogen metabolic wastes and ii) as pigments for signalling and screening^15,24^. While laboratory studies of individuals of known age indicate that pteridine levels increase linearly with age^25–27^ and are independent of sex^28^, pteridines are degradation products of purine metabolism and their accumulation may not be independent of physiological activity. Indeed, laboratory studies show pteridine levels to increase with rearing temperature^29,30^ and ambient light levels^31,32^.

A final difficulty in comparing the results of age-grading studies is that in addition to the biological variation outlined above, substantial differences exist between the protocols used. For example, many authors pooled the concentration of several pteridines in the eyes, even though their biosynthetic pathways are known to differ and they can convert into each other^33^. Protocols also differed with respect to whether pteridines were extracted only from eyes or the whole head. Further, the amount of light exposure during extractions^34,35^ and the duration of sample storage^36^ were often not mentioned. We feel that a systematic comparison of different approaches and protocols is useful and below provide such an overview. Based on this information, we experimentally test the effect of both biological as well as methodological conditions on the quantification of LC-MS/MS analyses of particular pteridines. Specifically, we examine the effects of the light-dark regime during extraction, extraction solvents and different insect body parts. We do so in groups of blood-sucking insects, bed bugs (*Cimex lectularius*) and bat bugs (*C. pipistrelle*), that vary in population genotype, diet and in rearing conditions.

## Results

### Review

The presence of specific pteridines in biological material was mentioned in 84 of 106 papers (79%), published between 1926 and 2019 (Figshare 10.6084/m9.figshare.11316473). These related to 44 different pteridines studied in 115 insect species from 32 families, however, some of them represented isomers (Supplementary Tables S1, S2). In 34% of publications the authors pooled pteridines, in 73% papers the authors worked with individual pteridines. At least one of the eight most intensely studied pteridines (6-biopterin, biopterin, erythropterin, isoxanthopterin, leucopterin, pterin, sepiapterin and xanthopterin) was mentioned in 67% of the publications. In 84% of the studied species, at least one of ten pteridines (8 mentioned above and two additional pteridines, i.e. 2-amino-4-hydroxypteridine and drosopterines) was described (Supplementary Table S1). Moreover, we found a lack of a consistent use of nomenclature for specific pteridines (Figshare 10.6084/m9.figshare.11316473). At least some of them are registered with the same CAS number (e.g. 492-10-4 with synonyms 7-methylxanthopterin and chrysopterine). This leads to that the same chemical compound was used under different trivial names in different studies (e. g. 2-amino-4-hydroxypteridine/pterin^37,38^, 7,8-dihydrobiopterin/deoxysepiapterin^16,39^, isoxantholumazine/violapterin^18,40^).

Previous studies listed 8 different extraction methods combined into at least 13 different protocols. The TRIS and ORG extraction solutions were used in 29 studies, hence other solutions were study-specific and used only a few times. In 75% of the papers, particular pteridines were compared against reference standards or literature. Commercially available standards were used in 61%, while in 39% of the studies, the extracted compounds were identified with the literature, i.e. comparing Rf values, spot colour or wavelengths. In 42.5% of studies, the samples were isolated in the dark or under red light. Isolation under reduced light intensity was mentioned in one study^34^ and 57% of studies did not specify any light conditions. Only one study tested how storage affected pteridine concentration^36^.

Only 39.6% (n = 42) of the papers focused on age-grading, while the majority of studies focused on the presence of different pteridines. These studies concerned 23 pteridines in 19 species from 15 families. In the context of age-grading, four analytical methods were commonly used, i.e. spectrofluorimetry (n = 35), HPLC (n = 8), thin layer chromatography (n = 5) and paper chromatography (n = 4). Pteridines for age-grading content were mainly extracted by TRIS (n = 15) and ORG (n = 14) solutions (see Methods). The most often studied body part was the head (n = 32).

### Optimization of LC-MS/MS method

#### Choice of extraction method

**Figure 1:**
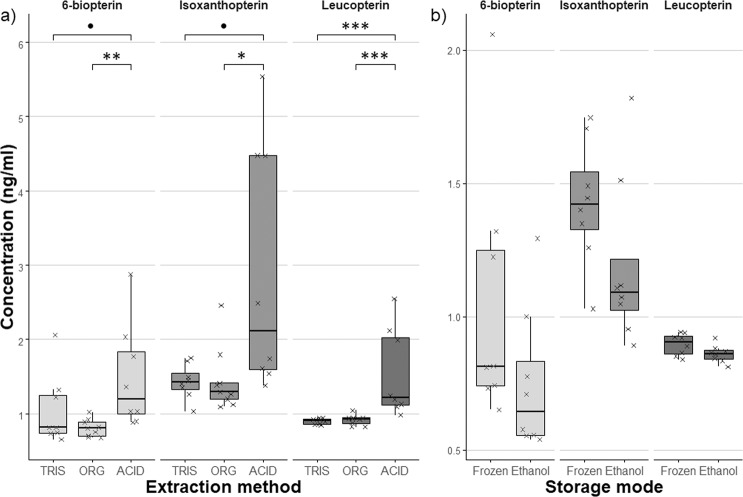
**(a**) Comparison of the extraction method efficiency. Data were tested by Kruskal-Wallis test. (**b**) Effect of biological material storage on the extracted pteridine concentration (8 replicants/storage mode). Individual measurements are illustrated by **×**. Data were compared with a two-sample t-value test. **‘’**: p > 0.1, **‘.’**: p < = 0.1, ‘*’: p < = 0.05, ‘**’: p < **=** 0.01, ‘***’: p < = 0.001.

There were significant differences in pteridine concentrations among extraction methods (Kruskal-Wallis test: *Bio*: H_2,26_ = 9.91, p = 0.007; *Iso*: H_2,26_ = 9.10, p = 0.011; *Leu*: H_2,25_ = 15.55, p < 0.001). Follow-up tests have demonstrated a significant difference between ACID and ORG method in all three studied pteridines (Fig. 1a). The difference in the pteridine concentration extracted by ACID and TRIS method was significant for *Leu* and nearly significant for *Bio* and *Iso*. TRIS and ORG method did not differ with either pteridine.

By method ACID we obtained the highest concentrations of pteridines but also the highest variation. The problem of ORG method was that two immiscible phases were produced after the addition of NaOH. Because the TRIS method showed no immiscible liquid phases and had a relatively small standard deviation, we used TRIS as the standard solvent for all experiments reported below.

#### Storage mode of biological material before extraction

There was no significant difference in pteridine concentration between samples frozen at −20 °C and stored in 70% ethanol extracted with the TRIS method (Fig. 1b, Supplementary Table S3).

#### Internal standard

**Figure 2:**
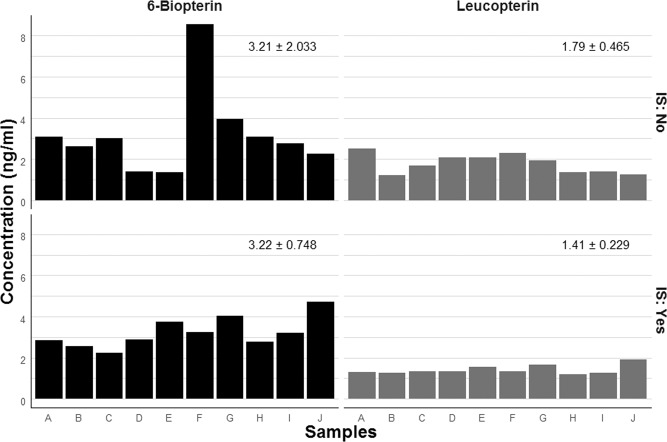
Comparison of measured concentrations of 6-biopterin and leucopterin of the same samples (n = 10) with and without correction for the internal standard 6,7-dimethyl-5,6,7,8-tetrahydropterine hydrochloride (means ± SD for each group). Pteridines were extracted by TRIS method.

IS reduced the variability of measured pteridine concentration because it removed the variability caused by the detection fluctuation of the machine during analyses (Fig. 2). The use of IS decreased variability by 10% for *Leu* (SD decreased from 0.465 to 0.229) and by 40% for *Bio* (SD decreased from 2.033 to 0.748). Because of that we used IS for all experiments reported below.

#### Stability of samples over time

The changes in the concentration of pteridines in the extract stored in the freezer for 14 days were up to 26% in samples spiked by pteridines mixture to a concentration 50 and 100 ng/mL and up to 36% in samples spiked to a concentration of 5 ng/mL (Table 1). However, the changes in concentrations are mostly non-significant (Table 1), the analyses of extract should be realized as soon as possible after extraction, to minimize errors in the analyses of individual analytes.

**Table 1:** Percentage of original pteridine content after 14 days (14D) in samples spiked on different concentration and results of paired t-test comparing the pteridine concentration in samples immediately after extraction and after 14 days stored in freezer.

Spiked concentration	6-biopterin			Isoxanthopterin			Leucopterin			Pterin		
	14D (%)	t	*p*	14D (%)	t	*p*	14D (%)	t	*p*	14D (%)	t	*p*
100 ng/mL	78.1	3.68	0.169	76.6	2.48	0.244	86.3	3.83	0.162	78.7	73.86	0.009
50 ng/mL	74.3	8.24	0.014	76.7	0.50	0.651	90.8	1.48	0.277	84.6	6.03	0.009
5 ng/mL	63.6	1.09	0.356	70.2	0.50	0.652	65.5	0.89	0.439	90.6	0.60	0.593

#### Accuracy

The accuracy of the LC-MS/MS method was evaluated as recovery in percent (RE%) of the amount of target analyte added into the sample. The average of all pteridine RE values for samples spiked to 100 ng/mL was 116%, for sample spiked to 50 ng/mL was 95%, for samples spike to 5 ng/mL was 96% (see more in Table 2). Whereas the standard error usually does not exceed the threshold of 5%, the variance of measured concentration is generally insignificant (Table 2).

**Table 2:** Comparison of the accuracy of the LC-MS/MS method for different spiked concentration represented by recovery percent of the known added amount of target pteridine (RE%) and the mean measured concentration with the percentage of standard error (SE).

Spiked concentration	6-biopterin		Isoxanthopterin		Leucopterin		Pterin		ALL
	RE%	SE (%)	RE%	SE (%)	RE%	SE (%)	RE%	SE (%)	RE%
100 ng/mL	121	9.7	113	2.2	108	3.0	121	2.6	116
50 ng/mL	107	10.4	97	3.2	85	10.2	91	4.3	95
5 ng/mL	111	2.7	94	5.0	117	9.7	64	4.0	96

#### Light condition during the pteridine extraction

**Figure 3:**
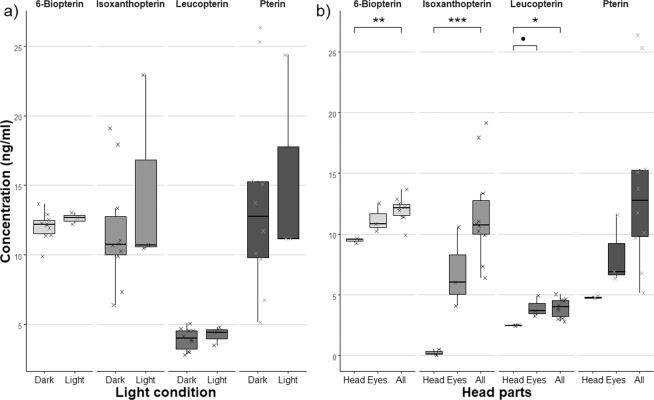
(**a**) Pteridine concentrations extracted under laboratory light conditions (Light, n = 3) and protected from light (Dark, n = 10). (**b**) Concentration of pteridines in whole head (All, n = 10) compared to separated head cuticle (Head, n = 3) and eyes (Eyes, n = 3). Individual measurements are illustrated by **×**. In 1^st^ experiments, data were tested by two-sample t-test and in 2^nd^ experiment by ANOVA (‘’: p > 0.1, ‘.’: p < = 0.1, ‘*’: p < = 0.05, ‘**’: p < = 0.01, ‘***’: p < = 0.001).

There was no significant difference in variance or the mean pteridine concentration (Fig. 3a) between protocols using extraction under normal laboratory light or dark conditions. Therefore, the more error-prone extraction in dark was not used.

#### Localization of pteridines in the head parts

Compared to whole head extracts, *Bio*, *Iso* and *Leu* were mostly stored in the eyes of bed bugs (ANOVA test: *Bio*: F_2,13_ = 8.25, p = 0.005; *Iso*: F_2,13_ = 11.73, p = 0.001; *Leu*: F_2,13_ = 4.73, p = 0.029) but *Pte* was clearly present also in the head cuticle (*Pte*: F_2,13_ = 3.09, p = 0.08) (Fig. 3b). The post-hoc tests revealed that this was due to a strong difference between the pteridine concentration of the head cuticle and of the whole head.

#### Head size effect on pteridine concentration

Head dimensions (eye width, head width, intraocular space dorsally) had no significant effect at all on pteridine concentration for *Bio*, *Iso* or *Leu* (t-test: all F_3,28_ < 0.01, all p > 0.05). Therefore, no body size correction was necessary.

#### Rearing conditions effect on the pteridine deposition

**Figure 4:**
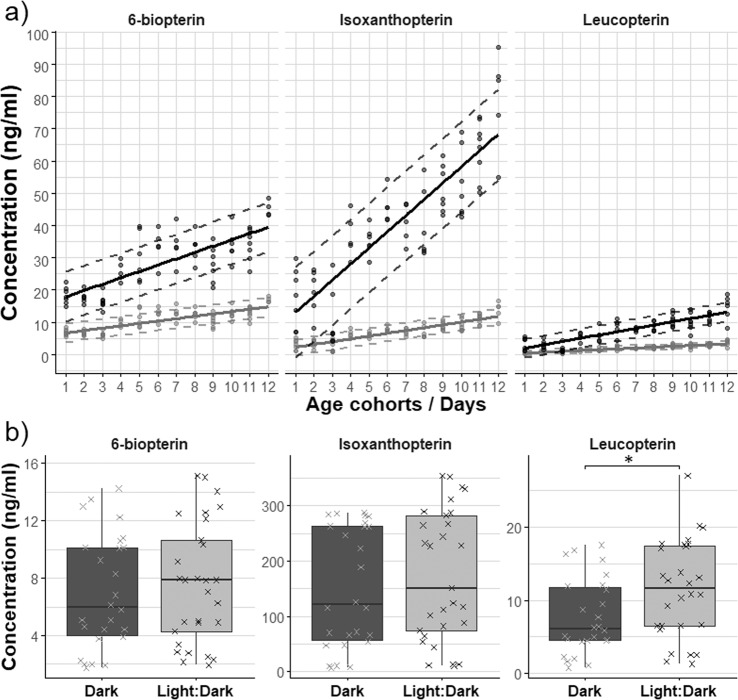
**(a**) Temperatures effect on pteridine accumulation over time. Bed bugs (BL, BAT1) were reared at 23 °C (grey) and at 27 °C (black). Dashed lines represents 80% prediction interval. Seven replicates per age cohort. Cohorts on x-axis: 1 (8-12 days), 2 (15–23 days), 3 (30–37 days), 4 (45–52 days), 5 (56–67 days), 6 (73–80 days), 7 (86–93 days), 8 (100–107 days), 9 (116–123 days), 10 (126–133), 11 (155–157), 12 (200–207), 13 (242–249). (**b)** Light-regime effect on pteridine accumulation. Bed bugs (HL, HUM2) were reared at 26 °C in dark (dark grey) and in 12 L/12D light regime (light grey). Individual measurements are illustrated by **×**. Data were tested by two-sample t-test (‘’: p > 0.1, ‘.’: p < =0.1, ‘*’: p < = 0.05, ‘**’: p < = 0.01, ‘***’: p < = 0.001).

We found a positive effect of temperature on the deposition of pteridines during the rearing of bed bugs (Fig. 4a). The light-regime had no significant effect on the deposition of *Bio* and *Iso* but a slight positive effect in 12 h/12 h L:D on *Leu* concentration (Fig. 4b).

#### Selection of pteridines for age-grading

Based on all previous methodical steps, *Iso* seems to be the most useful pteridine for age-grading in bed bugs. We excluded *Bio* because of large differences in the slope between BL populations (Table 3) and *Pte* based on different trends between lineages of *Clec* (Supplementary Figure S1). We also excluded *Leu*. Although *Leu* has positive trends in all studied populations, it could tend to overestimate the age due to a flatter slope of the regression curve (Table 3).

**Table 3:** Calibration curves, correlation coefficients and linear range of extracted pteridines in bugs head considering the origin of bugs. *C. lectularius*: BL represents pooled results for BAT1 and BAT2 populations, HL represents pooled results for HUM1 and HUM2. *C. pipistrelli*: *Cpip*.

Group	Regression equation	R^2^	min	max	H	p	n
*Clec BL*							
6-biopterin	y = 11.415 + 0.941×	0.07	5.2	48.4	22.43	0.033	147
Isoxanthopterin	y = 4.934 + 5.694×	0.73	3.9	101.8	111.42	<0.001	141
Leucopterin	y = 1.381 + 0.962×	0.66	0.8	18.6	105.71	<0.001	145
*BAT1*							
6-biopterin	y = 15.923 + 1.961×	0.59	13.0	48.4	53.71	<0.001	72
Isoxanthopterin	y = 7.971 + 5.015×	0.74	4.1	95.3	62.78	<0.001	74
Leucopterin	y = 0.952 + 1.023×	0.74	0.8	18.6	59.05	<0.001	74
*BAT2*							
6-biopterin	y = 5.517 + 0.242×	0.75	5.2	10.0	49.61	<0.001	75
Isoxanthopterin	y = 0.974 + 6.576×	0.43	3.9	101.8	52.11	<0.001	67
Leucopterin	y = 1.866 + 0.892×	0.58	1.1	15.4	53.53	<0.001	71
*Clec HL*							
6-biopterin	y = 16.105 + 0.276×	0.04	10.6	29.2	21.85	0.016	146
Isoxanthopterin	y = 22.706 + 2.077×	0.35	9.6	64.2	63.16	<0.001	143
Leucopterin	y = 2.406 + 0.975×	0.24	0.5	39.8	59.74	<0.001	147
*HUM1*							
6-biopterin	y = 13.468 + 0.300×	0.14	10.6	24.6	39.12	<0.001	77
Isoxanthopterin	y = 17.789 + 2.088×	0.53	9.6	50.2	51.50	<0.001	75
Leucopterin	y = 1.939 + 0.544×	0.42	0.5	14.4	50.12	<0.001	77
*HUM2*							
6-biopterin	y = 18.832 + 0.286×	0.05	12.0	29.2	38.05	<0.001	69
Isoxanthopterin	y = 27.674 + 2.144×	0.39	14.6	64.2	44.44	<0.001	68
Leucopterin	y = 2.623 + 1.523×	0.42	1.8	39.8	53.85	<0.001	70
*Cpip*							
6-biopterin	y = 3.816 + 0.194×	0.41	3.2	7.1	40.49	<0.001	60
Isoxanthopterin	y = 14.741 + 2.298×	0.47	9.0	48.5	38.93	<0.001	58
Leucopterin	y = 11.569 + 2.453×	0.58	8.9	45.9	42.13	<0.001	61

#### Verification of calibration curves with blind samples

Blind samples split into three age cohorts did not reveal significant differences in pteridine concentrations between 2nd and the other two age cohorts (Table 4). Therefore, we omitted the middle cohort and verified the correct grouping of blind samples from the youngest and the eldest cohort for all three pteridines separately (Table 5). When we compared the slopes of regression lines based on blind samples and calibration curves based on laboratory-reared bed bugs (see below “Assay of laboratory-reared *C. lectularius* and *C. pipistrelli* of known age”), it seemed that only the slopes of *Iso* were similar (Table 4), which reinforced our decision from the previous step to include *Iso*. When we split samples from the middle age cohort to the youngest or eldest age cohorts by K-means analysis, the presumption of increasing pteridine concentrations held (Mann-Whitney U test: *Bio*: Z = −2.57, *Iso*: Z = −2.76, *Leu*: Z = −3.06, all: p < 0.01).

**Table 4:** Comparison of slopes of regression lines based on laboratory-reared bed bugs (Lab.; HL, HUM2) and on blind samples (Blind; HL, HUM2) and results of comparison of individual age groups of blind samples.

Pteridin	Lab.	Blind	Slope			Blind Kruskal-Wallis test			Blind - Post-hoc t-tests		
			I vs. II			I vs. III			II vs. III		
			H	*p*	n	z′	*p*	z′	*p*	z′	*p*
6-biopterin	0.286	0.059	5.14	0.077	21	—	—	—	—	—	—
Isoxanthopterin	2.144	1.951	8.14	0.017	21	1.97	0.145	2.84	0.014	0.85	1.000
Leucopterin	0.523	0.181	11.74	0.003	20	1.32	0.563	3.38	0.002	1.97	0.147

**Table 5:** Proportion of blind samples (HL, HUM2) assigned to the correct 2 (rows 4–7) or 3 (rows 1–3) age-cohorts by pteridines according to K-means clustering analysis before and after excluding 5% outliers (Out).

Cohort	% correct		
	6-biopterin	Isoxanthopterin	Leucopterin
I (n = 9)	77.78	66.67	88.89
II (n = 10)	40.00	30.00	30.00
III (n = 11)	9.09	63.64	36.36
I (n = 9)	88.89	77.78	88.89
III (n = 11)	54.55	63.64	54.55
I_out_ (n = 8)	100.00	87.50	100.00
III_out_ (n = 10)	60.00	70.00	60.00

### Assay of laboratory-reared *C. lectularius* and *C. pipistrelli* of known age

We calculated calibration curves of *Iso* and *Leu* for *Clec* BL and *Clec* HL (by combining the results of the two appropriate populations) and for *Cpip* (Table 3). The concentration of both pteridines significantly and linearly increased with age for bugs of all populations, though in *Leu* with a lower slope (Table 3).

The concentration of both pteridines was strongly affected by age cohort (GLS: *Iso*: F_1,336_ = 394.10, *Leu*: F_1,347_ = 373.60, p < 0.001), and the linear relationship between concentration and age differed in slope between lineages (GLS: *Iso*: F_2,336_ = 50.26, *Leu*: F_2,347_ = 12.97, both p < 0.001). The *Iso* accumulation rate of BL significantly differed from that of HL (partial t-test: t_342_ = −9.48, p < 0.001) and *Cpip* (partial t-test: t_342_ = −7.71, p < 0.001) but not between HL and *Cpip* (partial t-test: t_342_ = 0.30, p > 0.05). *Leu* accumulation rate of *Cpip* was different from both lineages of *Clec* (partial t-test: BL: t_353_ = 5.09, HL: t_353_ = 21.09, p < 0.001) that did not differ from each other (partial t-test: t_353_ = 0.08, p > 0.05).

### Assay of field-sampled bed bugs *C. lectularius*

**Figure 5:**
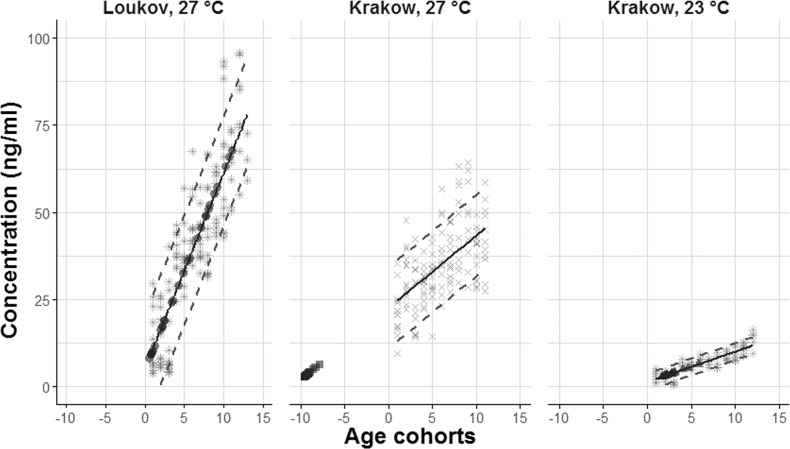
Predicted age of females from wild population based on isoxanthopterin. Calibration curves (solid lines) and 80% prediction intervals (dashed lines) based on laboratory reared samples (BL: ☼; HL×) for each lineage. Field samples are illustrated by • (BL, Loukov predicted by BL calibration curves at 27 °C), ■ (HL, Krakow predicted by HL calibration curves at 27 °C) and ▲ (HL, Krakow predicted by BL calibration curves at 23 °C).

Based on calibration curves from laboratory-reared *Clec* lineages (at 27 °C), individuals from Loukov (BL) varied in age by approximately 155 days. The calculated age cohorts varied from 0.6 to 11.1 (mean=4.7 cohort, n = 30). Krakow (HL) individuals differed by up to 2 age cohorts, i.e. 25 days, ranging from −9.6 to −7.8 age cohort (mean = −9.1 cohort, n = 20) (Fig. 5). The negative predicted age of Krakow females was almost certainly an effect of the high temperature in the laboratory model (27°C), which will not be reached in human settlements. Using the 23°C prediction curve from laboratory-reared *Clec* BL females at 23 °C, produced an age range of 4 cohorts (approx. 50 days) with a mean of 2.7 and ranging from 1.6 to 5.9 cohorts.

## Discussion

### Review

We identified that the literature seems to vary widely in the methods used to analyse pteridines, and this was true regardless of whether or not there was an application in age-grading. Variation especially existed in the method of pteridine extractions, the process of identification of different pteridine compounds and the use of internal standards.

In the studies we reviewed, we identified a lack of a consistent use of trivial nomenclature for specific pteridines. Older studies using paper chromatography as well as newer studies using more modern methods, especially in biological journals, used different trivial names for same chemical compound. Based on the literature review we have developed and here presented a reliable LC-MS/MS method to quantify four bioactive pteridines (*Bio*, *Iso*, *Leu* and *Pte*) simultaneously in bed bug heads. We also show this assay to be suitable to monitor pteridines in wild and laboratory-reared bed bugs. These results have implications for future researchers aiming to optimise their age determination protocols and for the application of the pteridine method to age-grade insects in the wild.

### Optimization of extraction, storage and LC-MS/MS methods

One of the first methodological complications one encounters is the extraction of pteridines from biological material. Alkaline or acid extractions are usually used because many pteridines are pH sensitive and decompose readily at a defined pH^41^. The use of very low pH conditions, usually pH ~ 3, can often lead to ring cleavage^42^, while below pH 8.8 *Iso* and *Leu* are eluted^43^. Our results showed that the ACID method, which was used for extraction in only two studies, provides the highest concentrations of pteridines, but with a high among sample variation in concentrations.

We illustrated that the usage of different extraction methods leads to large variability among samples and even the absence of some compounds after physical separation during liquid chromatography. Many authors solve this problem by jointly analysing more pteridines, but this way they do not reveal which compounds they have in a sample^36,44–46^. In such studies, the authors rely only on the fluorescence excitation wavelength of all analytes. In the studies where standards are not applied, the authors can only use the emission wavelengths, Rf values or colours from paper chromatography published for specific pteridines, which can be expected in their biological samples^47,48^. Therefore, the publication of pooled concentrations can lead to misinterpretation when evaluating the change in concentration over time, e.g. in age-grading. We confirm that the direct comparison of the wavelengths of the standards and peaks found in the samples, as included in most studies from the last two decades, is the most appropriate method (e.g. Tomic-Carruthers *et al*^16^., Tornero *et al*^49^.).

The extraction procedure is time-consuming and usually complicates the design of the experiments. In our study, the number of samples needed to produce calibration curves was impossible to dissect and measure within one day by one person. Therefore, we performed stability tests of pteridines in aqueous TRIS extracts comparing analyses of such extracts i) immediately after addition of known concentration of standards and ii) after 14 days storage of these samples in the freezer at −20 °C. Our results showed a decrease in concentrations of pteridines. This is consistent with previously published findings^50^. Therefore, the analyses of extracts should be realized as soon as possible after extraction. It seems that this recommendation has intuitively already been considered. Most studies (93% of researched papers) extracted new samples just before measurement and only in six studies samples were stored. If pre-analysis storage should be necessary, we recommend precise statements of the freezing conditions, in fact more precise than in current practice.

The light conditions of the sample analysis have been suggested a critical step in the extraction process^29^. Our results show that the extraction of pteridines requiring crushing the eye in a micro-tissue grinder in the dark was complicated and attracted a high error rate. Pteridines were below the detection limit or absent completely in many samples, caused by the unsuccessful disruption of the eye. Our successful dark extractions showed similar concentrations of *Bio*, *Iso* and *Leu* as extraction during laboratory light. Therefore, our results suggest instead that dark extraction may not be necessary^34^.

Pteridines can occur in three oxidation states with different stability^51^. Many authors mentioned different sensitivity in fluorescence detection of pteridines arising from their oxidation properties, among others also from the sensitivity to light^52,53^. At neutral pH, reduced forms are easily oxidized by molecular oxygen giving rise to stable fluorescent forms. A wide range of techniques (pre–treatments) and sample matrices are available to oxidize any residual reduced pteridines in order to facilitate the analysis by using more stable and more fluorescent, oxidized forms^49^. Most of the reviewed studies draw attention to pteridines with multiple oxidation states that are labile to heat, light, and auto-oxidation. The currently used published standard protocols^31,54^ should therefore, be augmented by more detailed description of the extraction methods.

### Distribution of pteridines in the body

Conventional wisdom has it that pteridines are found in the eyes only. We did not detect different pteridine concentrations in the eyes and whole head capsule, as the majority of pteridines was included in bed bug eyes. However, recently it was found that the pteridine biosynthesis pathway has been co-opted in the embryo to produce various pteridines in the antennae and legs^55^. This might explain non-significant difference of *Pte* between head cuticle and whole head capsule. It is possible, that generally the occurrence of pteridine in the specific parts of the body of the chosen model organism is important. Also, it is not always possible to dissect exactly the same amount of tissue from each individual. For example, eye preparation is complicated especially in small model organisms and extends the light exposure time. Therefore for the determination of differences in age-grading, we used only *Bio*, *Iso* and *Leu* stored in the eyes of bed bugs, while *Pte* are probably present also in the head cuticle.

We did not find higher pteridine concentrations in larger heads, similarly as Robson *et al.*^56^ for *Drosophila serrata*. Since the differences among bed bug cohorts are represented by the slope of the calibration curves, not by the positive/negative trend itself, an effect of the body size on the speed of pteridine accumulation cannot be excluded. In general, body size is connected to eye morphology, but the interaction between visual signalling and body size is not straight-forward; visual signalling species with smaller eyes showed a greater increase in maximum facet diameter than visual signalling species with larger eyes^57^. An increase in the facet diameter improves the eye’s sensitivity by improving photon capture^58^. Thus, differences in facet diameter within the wood ant compound eye are presumably connected with different requirements on the sensitivity and resolution of different eye regions when constrained by resources and space available^58^. Therefore, the morphology of the insect eye does not show a direct link between eye size, its function and pteridine accumulation.

### Other parameters affecting pteridine concentrations

Pteridines have a different accumulation rate with regard to several environmental factors, such as light, temperature, food etc.^59^ or physiological aspects, such as metabolic activity^60^. In our experiment, pteridine accumulation significantly increased with higher rearing temperatures. This positive effect of temperature on the pteridine accumulation has also been found in *Stomoxys calcitrans*^54^ and in *Musca domestica*^32^. Adult *Drosophila* developing at 27 °C showed greater head capsule fluorescence than adult flies developing at 17 °C^32^. These differences persisted for 15 days^32^.

Diet is another factor that can affect the concentration of pteridines in the eyes. For example, the changes in pooled concentrations of six pteridines showed a characteristic pattern of increased and/or decreased amounts in response to dietary levels^61^. Even though we did not directly test the effect of diet in our study, we would like to highlight the potential importance of diet when applying laboratory results to age-grading in wild populations. However, because the pteridine concentrations in *Cpip* (feeding on bat blood) were more similar to *Clec* HL (feeding on human blood) rather than *Clec* BL (that also feed on bat blood), it is clear that *Cimex* pteridine concentrations are not affected by bat or human blood *per se*.

### Age-dependence of pteridines in wild populations

It seems doubtful that pteridines are general indicators of chronological age, given their variation with temperature and between populations. Concentrations of pteridines usually correlate positively with the age of an individual^27,62^. However, first of all, it is unlikely that the deposition of pteridines is independent of metabolic activity. Second, the relationship with age does not apply to all pteridines. We showed that the concentration of *Iso* and *Leu* correlates positively with the age of the bed bugs (Table 3), while the concentration of *Pte* even decreased, but only in two HL populations and *Cpip* (Supplementary Figure S1). We only used females in our experiments but note that males and females can even show opposite patterns^63^. The concentrations of xanthopterin decreased markedly in the heads of males and females with increasing moth age, while those of *Pte* decreased more slowly and *Bio* remained largely unchanged in both sexes.

For two wild populations, we applied age-grading estimated based on concentrations of pteridines obtained from laboratory populations. The Loukov (BL) population, had a very large variations in age structure. That corresponds well to the fact that we not only found juveniles, but also very old females, which survived the winter in bat roosts. In contrast, for human-associated bugs (Krakow) we estimated a very low age, formally even reaching negative values. This finding can be expected for HL because bed bug control actions are likely to reduce average age in infestations. These actions are apparently not carried out in bat roosts. Bed bug individuals in human settlements can be expected to develop in temperatures less than 27 °C, the temperature of our calibration curves. Assuming a room temperature of 23 °C, easily expected in human dwellings, the negative age values disappear. A low age of a bedbug population is, of course, expected with bed bug control actions, which are not carried out in bat roosts.

Although the method of liquid chromatography of pteridines is often used for age-grading in insect, it still has restrictions. Our research showed that the calibration curves based on individuals bred under standard conditions are specific not only for taxonomic species, but also showed differences between populations, indicating other, yet unknown intrinsic factors that affect different pteridine accumulation between populations. In wild populations, we can expect even greater deviations, where the temperature and availability of food are significant factors as well^14,32^.

## Conclusion

We confirmed that after optimization of the extraction method, LC-MS/MS can be successfully used to determine the age in individual bed bugs based on different concentrations of pteridines. However, we recommend, before preparing the calibration curves, to thoroughly test the characteristics of the pteridine standards, the feasibility of methodical procedures, in particular the extraction of pteridines from biological material, and not to rely too much on completeness of published protocols. It is advisable to ensure that enough different standards are available to exclude pteridines that do not show the necessary trend among age cohorts of the model taxon. Our results indicate limits in the age-grading using pteridine concentrations in wild samples calibrated on the laboratory model. Based on pteridine concentrations estimating age with days accuracy is not possible, therefore we recommend working with age cohorts.

## Materials and methods

### Literature review

To identify the most commonly used and most suitable pteridines, we reviewed the existing body of literature using Google Scholar, Web of Science and the following keywords: pteridine AND insect AND method. This literature review yielded 139 studies but not all of them were based on suitable methodological steps. From the remaining 106 papers we conducted a detailed analysis of the pteridine extraction methods used and the pteridines found in insect (online Figshare dataset, 10.6084/m9.figshare.11316473).

### The study system

Bed bugs (*Cimex lectularius*) (*Clec)* are well-known human parasites that can also be associated with bats. Human-associated and bat-associated bed bugs, henceforth called human lines (HL) and bat lines (BL), are two separate genetic lineages that may eventually diverge into two different species^64^. We also examined a closely related species, *Cimex pipistrelli*^64,65^, henceforth called the bat bug, that is only associated with bats. Both species belong to the Cimicidae (Insecta: Hemiptera) and exclusively live on the blood of either bats or humans, are nocturnal. They can live for up to one year and experience different temperature ranges^12^.

### Insect origins and culture conditions

*Clec* BL bugs were collected at two nursery colonies of mouse-eared bats (*Myotis myotis*) roosting under roofs of the churches in Hanušovice and Raškov (North Moravia, Czech Republic), arbitrarily called BAT1 and BAT2. Bat bugs (*Cpip*) were collected from colonies of *M. myotis* roosting under the roof of the castle in Luhačovice (south Moravia, Czech Republic). All bugs were cultured in incubators at 23 ± 1 °C and 27 ± 1 °C in (see below) complete dark at 70% relative humidity. They were fed weekly on bat blood using the protocol of Wawrocka and Bartonička^66^. Bats were fed *ad libitum* with a mixed diet consisting of crickets (*Acheta* spp.) and mealworms (*Tenebrio molitor*) and, after experiments, returned to the colony. All the protocols are carried out according to relevant guidelines and regulations. Experimental procedures were approved by the Ethical Committee of the Masaryk University (No. 1/2018). All bats were captured, handled and temporarily kept in captivity under the licence issued by the South Moravian Regional Authority (Permit JMK 24451/2013 and 63761/2017). TB is authorised to handle free-living bats under the Certificate of Competency No. CZ01297 (§17, law No 246/1992), No. 922/93-OOP/2884/93 and 137/06/38/MK/E/07 of the Ministry of Environment of the Czech Republic.

*Clec* HL bugs were sourced from two lab cultures at Bayreuth University, Germany which were originally collected in Budapest, Hungary and London, United Kingdom, arbitrarily called HUM1 and HUM2. They were maintained in an incubator at 27 ± 1 °C in either complete darkness or a 12 h/12 h L:D light regime, at 70% relative humidity. They were fed weekly on human blood (conserved by CPDA; obtained from Faculty Hospital Bohunice, Brno with permission to use it for research purposes) using the protocol by Aak and Rukke^67^.

To control for age, newly eclosed adults of the respective populations were taken from the stock cultures for the experimental trials (for details see below “Assay of laboratory-reared *C. lectularius* and *C. pipistrelli* of known age”). Methodological steps were tested on same-aged adult females from BAT1. All samples were freeze-killed and stored at −20 °C until chemical extractions and analyses were conducted.

### Sample preparation

Bed bugs were decapitated and the head capsules extracted immediately using a microscalpel. Each bed bug head was homogenized separately in a glass 25–100 μL micro-tissue grinder (P-LAB, Czech Republic) with 50 μL of buffer and the suspension was transferred to a 1.5 mL Eppendorf vial. To ensure that all parts of the head capsule were removed and to maximize the amount of transferred pteridine, 50 μL of buffer were added three times into the micro-tissue grinder and transferred to the suspension. The amount of buffer in the vial was supplemented according to the extraction method TRIS^29^ or ORG^31^ or ACID^68^ (see Supplementary methods). Vials with a suspension were left standing in the ultrasound for approximately 2 hours. During this period, the ultrasound was turned on three times for 15 min (at the beginning, after 1 hour and at the end). After that, the suspension was centrifuged for 5 min at 6000 r.p.m. The 0.5 mL of supernatant was transferred into a sealed dark glass vial and stored at −20 °C until the LC-MS/MS analysis was conducted. The head capsule was homogenized and transferred under normal light conditions, all other extraction steps were conducted in the dark.

### Pteridine standards

Based on our literature review (see Results), we chose 6-biopterin, isoxanthopterin, leucopterin and pterin as standards for liquid chromatography-tandem mass spectrometry (LC-MS/MS). They were selected from 44 potential pteridines (Supplementary Table S1) because they were tested on the largest number of insect families. Moreover, isoxanthopterin and leucopterin were found in other families of Heteroptera and we expected to be present in bed bugs as well. Erythropterin was excluded because no manufacturer of the standard was found. The name of 6-biopterin was used instead of simple biopterin due to the same CAS number. For optimization of methodological steps and to establish calibration curves for age-grading we used 6-biopterin (*Bio*, CAS number: 22150–76–1) and isoxanthopterin (*Iso*, CAS 529-69-1) obtained from Sigma Aldrich Corporation (St Louis, MO, U.S.A.), leucopterin (*Leu*, CAS 492-11-5) and pterin (*Pte*, CAS 2236-60-4) from Schircks Laboratories (Switzerland).

The stock solutions of pteridine standards were prepared by dissolving 4 mg of each pteridine in 10 mL water solution comprising 0.1% (w/v) dithiothreitol and 40 mmol/L ammonium hydroxide (DTT solution). Then, we mixed appropriate volume of each pteridine stock solution and DTT solution to obtain a concentration of 10 µg/mL. From this mixed stock solution, we prepared calibration standards by serial dilution in acetonitrile.

### LC-MS/MS analysis

Liquid chromatograph Agilent 1290 Infinity II Series (Agilent Technologies, Santa Clara, CA was used for separation of mixture of target pteridines. Separation was realised using the chromatographic column Luna NH_2_ (100 Å 150 ×2.0 mm 3μm, Phenomenex, USA) at column temperature 30 °C; the volume injected sample was 5 µl^69^. Mobile phase for binary gradient elution consisted from (A): twice demineralized water containing 0.1% (v/v) formic acid and 10 mmol/L ammonium acetate and (B): acetonitrile containing 0.1% (v/v) formic acid. The gradient elution started at 15% (A) + 85% (B) and changed in 0-5 min interval of analyses from 85% (B) to 80% (B). Then in 5-6 min interval changed from 80% (B) to 85% (B) with a subsequent equilibration step of 85% (B) to 11 min. The flow rate of mobile phase was 0.4 mL/min. The Agilent 6495 Triple Quadrupole (Agilent Technologies, Santa Clara, CA) operating in the ESI-negative SRM mode was used for MS/MS analysis. Two MS/MS transitions of each pteridine were used for quantitative LC-MS/MS analyses. The conditions of triple quadrupole instrument optimised for analyses of pteridines were as follows: drying gas temperature 290 °C, drying gas flow 11 L/min, nebulizer gas pressure 45 psi, sheath gas temperature 350 °C, sheath gas flow 10 L/min, capillary voltage 3 500 V^70^. SRM transition of MS/MS analyses of pteridines are summarized in Table 6. The fragment ion with the highest abundance of each pteridine precursor was used for the quantification of the respective analyte. The fragment ion with the second highest abundance was used to confirm the presence of the respective analyte.

**Table 6:** SRM transitions of pteridines analysed by LC-MS/MS method.

Compound	t_R_ [min]	Precursor ion m/z	Product ion m/z
DMTH Pterin	2.3	190.1	172.1
		190.1	147.1
Pterin	3.2	162.2	144.1
		162.2	119.0
Isoxanthopterin	4.1	177.9	136.0
		177.9	161.1
6-biopterin	4.4	236.0	192.1
		236.0	146.9
Xanthopterin	4.5	177.9	160.0
		177.9	135.0
Leucopterin	5.3	193.9	166.1
		193.9	149.0

A method of internal standard calibration was used for the final quantification of the target compounds in the respective extract. Calibration curves were constructed in the concentration range 1.0-100 ng/mL of each pteridine using an internal standard of 6,7-dimethyl-5,6,7,8-tetrahydropterine hydrochloride at a concentration of 30 ng/mL. Instrumental limits of quantification (mean LOQ = 5 µg per injection) were determined from the concentration of individual standards giving an analyte to noise ratio of MS/MS detection of 10:1.

Individual steps in optimization of LC-MS/MS method are described in supplementary materials (section S1).

### Assay of laboratory-reared *C. lectularius* and *C. pipistrelli* of known age

Using the optimized LC-MS/MS protocol, we determined the association between the pteridine concentration, and the female age in bed/bat bugs that differ genetically (i.e., population, lineage, or species). We examined the five stock populations described above (2 *Clec* BL and HL populations each, reared at 27 °C, and one population of *Cpip* reared at 23 °C). Seven females were taken at 11–13 time points (cohorts) after eclosion till day 249. Sampling was spaced from 7 to 17 days, the last two (oldest) cohorts were 42 days apart.

### Assay of field-sampled bed bugs *C. lectularius*

To test the age distribution in the wild, we obtained snapshot (one time point) samples from two populations that did not belong to the study populations. In June 2018, *Clec* BL females were collected in a bat nursery colony in Loukov (northeast Bohemia, Czech Republic; n = 30) and in May 2011, *Clec* HL was collected in an apartment building in Krakow (Poland; n = 20). To predict the ages of field-sampled females, the marginal models of pteridine concentration of *Clec* lineages reared at 27 °C

1 $$(Concentratio{n}_{ij}={\alpha }_{i}+\beta Cohor{t}_{j}+{\varepsilon }_{ij},)$$

were modified to form the predictive equations for individual age of wild bed bugs from different lineages (Table 3).

### Statistical analysis

All statistical analyses were performed using R 3.6.1^71^ using the packages *nlme*^72^ and *ggplot2*^73^, only K-means clustering analysis was made in STATISTICA^74^.

All data were tested for normal distribution using Shapiro-Wilk test and for homogeneity of variances using Levene’s test. In order to determine the extraction method efficiency, a Kruskal-Wallis test with following non-parametric post-hoc comparisons were performed. The difference between storage modes of biological material before extraction, the light effect during extraction, the temperature and light-regime effect during rearing were tested using two-sample t-tests. The importance of internal standard use was verified by Wilcoxon matched-pairs signed-ranks test. The sample stability over time was tested by paired t-test. To detect the localization of pteridines in head parts and head size effect on pteridine concentration, one-way ANOVA with post-hoc comparisons using Scheffé's method was used.

To verify the established calibration curves, pteridine concentrations of blind samples were grouped based on K-means clustering analysis (the initial cluster centres were computed using the method of sorting distances and taking observations at constant intervals). The relation between age and pteridine concentration in blind samples was described using a linear regression model and the differences between age cohorts were tested by Kruskal-Wallis test with the following non-parametric post-hoc comparisons or with Mann-Whitney U test.

The association between pteridine concentration and female age was conducted using general least squares (GLS) regression and a partial t-test testing homogeneity of the slope:

2 $$Concentratio{n}_{ijk}=\alpha +Lineag{e}_{k}+{\beta }_{k}Cohor{t}_{j}+{\varepsilon }_{ijk},$$

where age cohort (Cohort) was treated as a continuous variable, lineage (Lineage) was a fixed factor, subscripts *i*, *j* and *k* refer to lineage and age cohort of the particular individual, α represented the intercept of pteridine concentration, β the slope of the relationship between age and pteridine concentration and ε residuals. Lineage was replaced by population to test differences between populations.

## Supplementary information

Supplementary Information.

## Data Availability

All relevant data are within the paper as Supporting Information files (Tables S1-3 and Figure S1) and in dataset repository Figshare 10.6084/m9.figshare.11316473.
