# Supplementary material for: Identification and age-dependence of pteridines in bed bugs (Cimex lectularius) and bat bugs (C. pipistrelli) using liquid chromatography-tandem mass spectrometry
Source: Sci Rep. 2020 Jun 23;10:10146. doi: 10.1038/s41598-020-66919-5 (PMC7311437; doi:10.1038/s41598-020-66919-5)
Supplement: Supplementary file 1 — Supplementary Information. [file 41598_2020_66919_MOESM1_ESM.doc]

# Identification and age-dependence of pteridines in bed bugs (*Cimex lectularius*) and bat bugs (*C. pipistrelli*) using liquid chromatography-tandem mass spectrometry

Jana Křemenová^1*^, Ondřej Balvín^2^, Oliver Otti^3^, Michal Pavonič^1^, Klaus Reinhardt^4^, Zdeněk Šimek^5^, Tomáš Bartonička^1^

^1^Masaryk University, Faculty of Sciences, Department of Botany and Zoology, Brno, 61137, Czech Republic

^2^ Czech University of Life Sciences Prague, Faculty of Environmental Science, Department of Ecology, Prague, 16521, Czech Republic

^3^ Universität Bayreuth, Animal Ecology I, Animal Population Ecology, Bayreuth, 95440, Germany,

^4^ Technische Universität Dresden, Department of Biology, Applied Zoology, Dresden, 01069, Germany,

^5^ Masaryk University, Research Centre for Toxic Compounds in the Environment, Brno, 62500, Czech Republic

***Corresponding author**: Jana Křemenová, *Department of Botany and Zoology, Masaryk University, Kotlářská 2, 611 37 Brno, Czech Republic,* kremenoj@gmail.com, tel: +420 549493095

# Supplementary Methods

## Optimization of LC-MS/MS method

1. **Choice of extraction method**

Three types of extractions were tested for preparations of samples for LC-MS/MS: i) TRIS – 1 mL of 0.05M tris/HCL buffer at pH 8 (modified procedure from Mail *et al.*^1^; n_TRIS_=8); ii) ORG – 0.5 mL of 2:1 chloroform/methanol and after 1^st^ round ultrasound (see Sample preparation in Materials and methods in the main text) add 0.75 mL of 0.1M NaOH adjusted to pH 10 with glycine (modified procedure from Lehane *et al.*^2^; n_ORG_=9, ); iii) ACID – 1 mL of 4:1:5 methanol/acetic acid/water (modified procedure from Ferré *et al.*^3^; n_ACID_=9).

1. **Storage mode of biological material before extraction**

The two most common methods of biological material conservation, i.e. 70% ethanol and freezing at -20 °C, were tested to determine their influence on the extracted amount of pteridines (n = 8/treatment).

1. **Internal standard**

To minimize the effect of instrument error during LC-MS/MS analysis of individual samples, we used 6,7-dimethyl-5,6,7,8-tetrahydropterine hydrochloride as an internal standard (IS). Samples (n=10) were measured without IS and, subsequently, with addition 5 μL of 30 ng/mL of IS to each sample. According to the difference between the expected and measured MS/MS signal of IS we could correct the deviations in measured concentrations of pteridines caused by the instrument.

1. **Stability of samples over time and accuracy**

Due to the generally known low stability of pteridines in aqueous solutions, the stability of pteridines in analyzed TRIS extracts was tested within 2 weeks of storage in the dark in a freezer using the set of TRIS extracts spiked by pteridines in the concentration range of 5-100 ng/mL (n = 4/spiked concentration). Samples were analyzed using a calibration curve constructed from freshly prepared calibration solutions.

The accuracy of the LC-MS/MS method using IS was reported as the percent recovery (RE%) of the known added amount of target analyte determined by replicate analysis using analyses of 4 spiked samples (same as for stability test) at each of 3 concentration levels in the range of the calibration curve (similarly as Bílková *et al.*^4^).

1. **Light condition during the pteridine extraction**

In the literature, we found several papers mentioning that pteridines need to be protected from the light because they are light-sensitive^5–7^. Ten samples were prepared in the dark and three samples under normal light to test the light effect on the extracted amount of pteridines.

1. **Localization of pteridines in head parts**

To identify their main storage site, we extracted pteridines separately from eyes (n=3 pairs) and the rest of the head cuticle (n=3) and compared the concentrations to those obtained from whole heads (n=10).

1. **Head size effect on pteridine concentration**

Body size reflects in head size, but it is unknown whether this will be related to age-dependent pteridine concentration. Therefore, before the extraction, we took pictures of the bed bugs (n=8) using a microscope Keyence VHX-S550E and measured three head dimensions (eye width, head width, intraocular space dorsally) as detailed in Balvín *et al.*^8^.

1. **Rearing conditions effect on the pteridine deposition**

To test whether temperature has an effect on the deposition of pteridines during rearing, BAT1 females (*Clec* BL) were reared at either 23 °C or 27 °C. We collected individuals (n = 7/cohort) at twelve time points (cohorts) after eclosion till day 207. Cohorts were even spread by 7 to 17 days, the last (oldest) cohort collection time-point was separated by 50 days from the previous one.

Light conditions during rearing were tested on HUM1 (*Clec* HL) females. They were reared under either 24h darkness or a 12h/12h L:D light cycle (n = 30/treatment). They were collected at three time points, 23, 34 and 62 days after eclosion. Note that both light conditions are realistic - complete darkness for caves and closed buildings for bat-associated bugs, an alteration of L:D for human-associated bugs and more open buildings.

1. **Verification of calibration curves with blind samples**

Verification was done using the HUM1 (*Clec* HL) population. Three age cohorts were reared and sampled in the Bayreuth laboratory and sent to Brno where LC-MS/MS measurements and data evaluation were done blind with regard to the sample age (n = 10/cohort). This methodological step assumes an increase in pteridine concentration with age based on knowledge from “Assay of laboratory-reared *C. lectularius* and *C. pipistrelli* of known age“.

# Supplementary tables

**Table S1** List of all used (All) and age-grading focused (Age) pteridines, numbers of insect taxa in researched studies (publications).

| **Pteridines** | **Species** | | **Order** | | **Family** | | **Publications** | |
| --- | --- | --- | --- | --- | --- | --- | --- | --- |
|  | **All** | **Age** | **All** | **Age** | **All** | **Age** | **All** | **Age** |
| 2,4-diamino-6-hydroxymethyl-pteridine | 1 | 1 | 1 | 1 | 1 | 1 | 1 | 1 |
| 2-amino-4-hydroxypteridine | 52 | 2 | 7 | 2 | 11 | 2 | 10 | 3 |
| 2-Amino-6-oxy-pterin | 1 | - | 1 | - | 1 | - | 1 | - |
| 6-acetyl-7,8-dihydropterin | 1 | - | 1 | - | 1 | - | 1 | - |
| 6-biopterin | 6 | 6 | 3 | 3 | 6 | 6 | 6 | 6 |
| 6-propionylpterin | 1 | - | 1 | - | 1 | - | 1 | - |
| 6-pterincarboxylic acid | 8 | 3 | 3 | 1 | 6 | 3 | 8 | 4 |
| 7,8-dihydro-6-formylpterin | 1 | - | 1 | - | 1 | - | 1 | - |
| 7,8-dihydrobiopterin | 2 | 1 | 1 | 1 | 2 | 1 | 2 | 1 |
| 7,8-dihydroxanthopterin | 2 | 1 | 2 | 1 | 2 | 1 | 2 | 1 |
| 7-methylxanthopterin | 9 | 9 | 2 | 2 | 4 | 4 | 9 | 9 |
| Acetyldihydrohomopterin | 1 | - | 1 | - | 1 | - | 1 | - |
| Aminopterin | 1 | 1 | 1 | 1 | 1 | 1 | 1 | 1 |
| Aurodrosopterin | 1 | - | 1 | - | 1 | - | 1 | - |
| Biopterin | 48 | 4 | 4 | 3 | 12 | 4 | 18 | 4 |
| Deoxysepiapterin | 2 | 1 | 1 | 1 | 2 | 1 | 2 | 1 |
| D-neopterin | 1 | 1 | 1 | 1 | 1 | 1 | 1 | 1 |
| Drosopterins | 34 | 1 | 1 | 1 | 3 | 1 | 10 | 1 |
| Ekapterin | 1 | - | 1 | - | 1 | - | 1 | - |
| Erythropterin | 31 | 5 | 2 | 2 | 12 | 3 | 21 | 4 |
| Formicapterin | 1 | - | 1 | - | 1 | - | 2 | - |
| Guanopterin | 1 | - | 1 | - | 1 | - | 1 | - |
| Isodrosopterin | 2 | - | 1 | - | 2 | - | 2 | - |
| Isosepiapterin | 2 | - | 1 | - | 2 | - | 3 | - |
| Isoxantholumazine | 1 | 1 | 1 | 1 | 1 | 1 | 2 | 1 |
| Isoxanthopterin | 89 | 10 | 8 | 4 | 26 | 8 | 47 | 11 |
| Lepidopterin | 1 | - | 1 | - | 1 | - | 1 | - |
| Leucopterin | 26 | 4 | 2 | 2 | 10 | 2 | 21 | 3 |
| L-monapterin | 1 | 1 | 1 | 1 | 1 | 1 | 1 | 1 |
| L-sepiapterin | 4 | - | 1 | - | 3 | - | 1 | - |
| Luciopterin | 1 | 1 | 1 | 1 | 1 | 1 | 1 | 1 |
| Lumazin | 1 | - | 1 | - | 1 | - | 1 | - |
| Mesopterin | 3 | - | 1 | - | 1 | - | 2 | - |
| Neoprosopterin | 2 | - | 1 | - | 2 | - | 2 | - |
| Neopterin | 5 | 2 | 4 | 2 | 5 | 2 | 6 | 2 |
| Pteridin-7-carbonic acid | 1 | - | 1 | - | 1 | - | 1 | - |
| Pterin | 18 | 6 | 4 | 2 | 11 | 5 | 18 | 6 |
| Sepialumazine | 1 | 1 | 1 | 1 | 1 | 1 | 1 | 1 |
| Sepiapterin | 44 | 4 | 3 | 2 | 8 | 3 | 18 | 5 |
| Tetrahydrobiopterin | 1 | - | 1 | - | 1 | - | 1 | - |
| Tetrahydropterin | 3 | - | 1 | - | 3 | - | 2 | - |
| Violapterin | 5 | - | 2 | - | 5 | - | 3 | - |
| Xanthopterin | 41 | 6 | 7 | 5 | 18 | 6 | 35 | 7 |
| Y-pterin | 3 | - | 1 | - | 1 | - | 1 | - |

**Table S2** List of 32 insect families studied in context of pteridines (All) and focused on age-grading (Age).

| **Family** | **Species** | | **Pteridines** | | **Publications** | |
| --- | --- | --- | --- | --- | --- | --- |
|  | **All** | **Age** | **All** | **Age** | **All** | **Age** |
| *Blattodea* |  |  |  |  |  |  |
| Blattidae | 1 | - | 3 | - | 1 | - |
| *Coleoptera* |  |  |  |  |  |  |
| Tenebrionidae | 1 | - | 1 | - | 1 | - |
| Lampyridae | 1 | 1 | 1 | 1 | 1 | 1 |
| *Diptera* |  |  |  |  |  |  |
| Calliphoridae | 3 | 1 | 8 | 3 | 5 | 1 |
| Culicidae | 5 | 1 | 12 | 10 | 5 | 1 |
| Drosophilidae | 33 | 1 | 20 | 1 | 14 | 1 |
| Muscidae | 3 | 2 | 5 | 4 | 3 | 2 |
| Tephritidae | 2 | 1 | 8 | 7 | 4 | 2 |
| *Hemiptera* |  |  |  |  |  |  |
| Aphididae | 2 | - | 4 | - | 1 | - |
| Cercopidae | 1 | - | 3 | - | 1 | - |
| Cicadidae | 1 | - | 2 | - | 1 | - |
| Reduviidae | 1 | - | 3 | - | 1 | - |
| Scutelleridae | 10 | - | 6 | - | 2 | - |
| *Heteroptera* |  |  |  |  |  |  |
| Coreidae | 3 | - | 3 | - | 1 | - |
| Lygaeidae | 1 | 1 | 7 | 7 | 3 | 3 |
| Pentatomidae | 6 | - | 6 | - | 4 | - |
| Pyrrhocoridae | 5 | 3 | 9 | 6 | 8 | 3 |
| Rhopalidae | 2 | - | 4 | - | 2 | - |
| *Hymenoptera* |  |  |  |  |  |  |
| Apidae | 1 | 1 | 7 | 1 | 4 | 1 |
| Formicidae | 4 | 1 | 8 | 1 | 5 | 1 |
| Pteromalidae | 1 | 1 | 3 | 3 | 1 | 1 |
| Vespidae | 1 | 1 | 5 | 1 | 3 | 1 |
| *Lepidoptera* |  |  |  |  |  |  |
| Bombycidae | 1 | 1 | 3 | 3 | 1 | 1 |
| Gelechiidae | 1 | 1 | 4 | 4 | 1 | 1 |
| Lycaenidae | 1 | - | 5 | - | 1 | - |
| Nymphalidae | 2 | - | 2 | - | 2 | - |
| Papilionidae | 2 | - | 3 | - | 1 | - |
| Pieridae | 13 | 2 | 14 | 5 | 16 | 2 |
| Pyralidae | 2 | - | 6 | - | 7 | - |
| *Neuroptera* |  |  |  |  |  |  |
| Ascalaphidae | 1 | - | 1 | - | 1 | - |
| *Orthoptera* |  |  |  |  |  |  |
| Acrididae | 3 | - | 2 | - | 3 | - |
| *Phasmatodea* |  |  |  |  |  |  |
| Lonchodidae | 1 | - | 3 | - | 1 | - |

**Table S3** Comparison of efficiency of extraction methods with and without the correction for the different head dimensions. Measured concentrations are in ng/ml.

| **Extraction method** | **Storage mode** | **N** | **Mean** | **Min.** | **Max.** | **SD** |
| --- | --- | --- | --- | --- | --- | --- |
| *6-biopterin* |  |  |  |  |  |  |
| ACID | -20 °C | 9 | 1.949 | 0.883 | 5.662 | 1.540 |
| ORG | -20 °C | 9 | 0.807 | 0.677 | 1.020 | 0.117 |
| TRIS | -20 °C | 8 | 1.044 | 0.651 | 2.058 | 0.476 |
| TRIS | 70% ethanol | 8 | 0.751 | 0.539 | 1.294 | 0.270 |
| *Isoxanthopterin* | |  |  |  |  |  |
| ACID | -20 °C | 9 | 2.827 | 1.380 | 5.532 | 1.567 |
| ORG | -20 °C | 9 | 1.446 | 1.092 | 2.458 | 0.433 |
| TRIS | -20 °C | 8 | 1.429 | 1.030 | 1.746 | 0.232 |
| TRIS | 70% ethanol | 8 | 1.191 | 0.892 | 1.821 | 0.314 |
| *Leucopterin* |  |  |  |  |  |  |
| ACID | -20 °C | 9 | 1.856 | 0.979 | 4.424 | 1.110 |
| ORG | -20 °C | 9 | 0.915 | 0.820 | 1.045 | 0.069 |
| TRIS | -20 °C | 8 | 0.896 | 0.839 | 0.942 | 0.041 |
| TRIS | 70% ethanol | 8 | 0.861 | 0.813 | 0.920 | 0.032 |

# Supplementary figures

**Figure S1** Predicted age of females from laboratory reared populations based on pterin. Calibration curves (solid lines) and 80% prediction intervals (dashed lines) (● *Clec* BL; ■ *Clec* HL; ▲ *Cpip*) for each lineage.

**
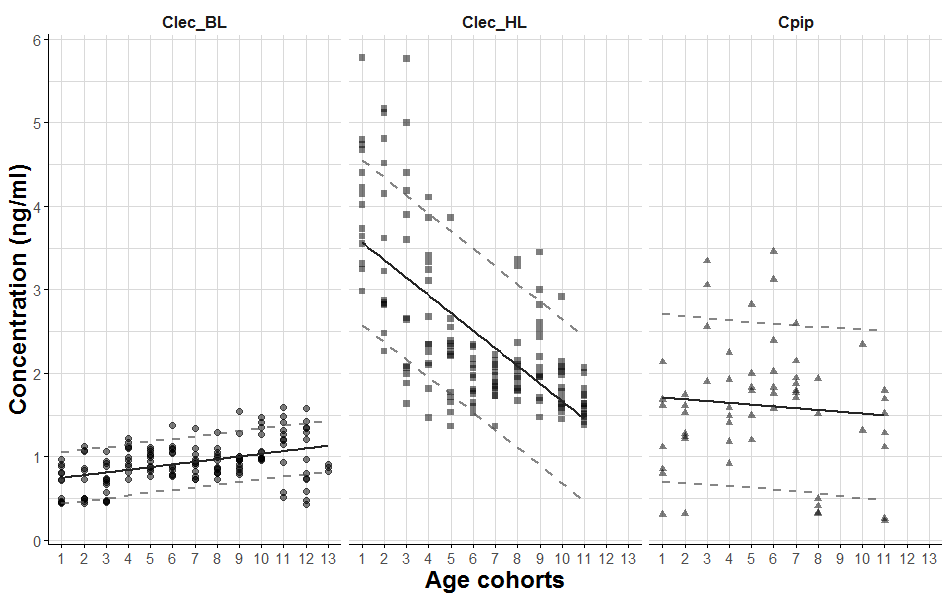
**

# References

1. Mail, T. S., Chadwick, J. & Lehane, M. J. Determining the age of adults of Stomoxys calcitrans (L.) (Diptera: Muscidae). *Bull. Entomol. Res.* **73**, 501 (1983).

2. Lehane, M. J. & Mail, T. S. Determining the age of adult male and female *Glossina morsitans* morsitans using a new technique. *Ecol. Entomol.* **10**, 219–224 (1985).

3. Ferré, J., Silva, F. J., Real, M. D. & Ménsua, J. L. Comparative study of the eye colour mutants of *Drosophila melanogaster*: quantification of the eye-pigment and related metabolites. in *Chemistry and Biology of Pteridines: Proceedings* (eds. Kisliuk, R. L. & Brown, G. M.) 669–673 (Elsevier/North-Holland, 1979).

4. Bílková, Z., Adámková, M., Albrecht, T. & Šimek, Z. Determination of testosterone and corticosterone in feathers using liquid chromatography-mass spectrometry. *J. Chromatogr. A* **1590**, 96–103 (2019).

5. Ziegler, I. & Harmsen, R. The biology of pteridines in insects. *Adv. In Insect Phys.* **6**, 139–203 (1970).

6. Pfleiderer, W. Pteridines. in *Comprehensive Heterocyclic Chemistry* 263–327 (Elsevier, 1984). doi:10.1016/B978-008096519-2.00038-2

7. Albert, A. The Pteridines. in *Fortschritte der Chemie Organischer Naturstoffe / Progress in the Chemistry of Organic Natural Products / Progrés dans la Chimie des Substances Organiques Naturelles* 350–403 (Springer Vienna, 1954). doi:10.1007/978-3-7091-8014-3_8

8. Balvín, O., Munclinger, P., Kratochvíl, L. & Vilímová, J. Mitochondrial DNA and morphology show independent evolutionary histories of bedbug Cimex lectularius (Heteroptera: Cimicidae) on bats and humans. *Parasitol. Res.* **111**, 457–469 (2012).
